# Association between Intimate Partner Violence and Contraceptive Use Discontinuation in India

**DOI:** 10.1111/sifp.12184

**Published:** 2022-01-15

**Authors:** Ashish Kumar Upadhyay, Kaushalendra Kumar, K. S. James, Lotus Mcdougal, Anita Raj, Abhishek Singh

**Affiliations:** ^1^ Research Coordinator, GENDER Project International Institute for Population Sciences Mumbai 400 088 India; ^2^ Assistant Professor, Department of Public Health & Mortality Studies International Institute for Population Sciences Mumbai 400 088 India; ^3^ Director and Senior Professor International Institute for Population Sciences Mumbai 400 088 India; ^4^ Associate Project Scientist, Center on Gender Equity and Health University of California San Diego La Jolla CA 92093 USA; ^5^ Tata Chancellor Professor of Medicine and Director, Center on Gender Equity and Health University of California San Diego La Jolla CA 92093 USA; ^6^ Professor, Department of Public Health & Mortality Studies International Institute for Population Sciences Mumbai 400 088 India

**Keywords:** intimate partner violence, contraceptive use discontinuation, contraceptive use discontinuation while still in need (DWSIN), 2015–2016 National Family Health Survey (NFHS‐4), India

## Abstract

Research on the association between experiences of intimate partner violence (IPV) and contraceptive use discontinuation in low‐ and middle‐income countries (LMICs) is limited. This study aims to fill this important gap using microdata collected from women aged 15–49 in the 2015–2016 National Family Health Survey (NFHS). Analyses used multivariable multinomial logistic regressions stratified by long‐acting reversible contraceptive methods (LARC)/non‐LARC and condom/pill to examine the association between experience of IPV and contraceptive use discontinuation while still in need (DWSIN). Experience of physical violence was associated with DWSIN among LARC/IUD users (RRR: 3.73, 95 percent CI [1.55–8.95]) Among condom users, DWSIN was higher among women who experienced emotional violence compared with women who did not experience any violence (RRR: 4.16, 95 percent CI [1.59–10.90]). Although we did not find an association between IPV and overall contraceptive use discontinuation, we did find compelling evidence of an association between IPV and IUD and condom use discontinuation in India. There is a need to understand women's experience of IPV as a part of a broader strategy to provide high‐quality family planning services to all women while considering individual circumstances and reproductive aspirations to support the uninterrupted use of contraception in India.

## BACKGROUND

Recent estimates from the World Health Organization (WHO) indicate that more than one in three women (35 percent ) experienced either physical and/or sexual intimate partner violence (IPV), or nonpartner sexual violence in their lifetime (García‐Moreno et al., 2013). A majority of this violence is perpetuated by an intimate partner. Globally, about 30 percent of women experienced physical/sexual violence by their intimate partner(s) in their lifetime. Recent estimates from the 2015–2016 National Family Health Survey (NFHS‐4) show that about 31 percent of ever‐married women in India have experienced spousal physical, emotional, or sexual violence in their lifetime (IIPS and ICF 2017). Spousal physical violence was most prevalent (27 percent), followed by emotional violence (13 percent), and sexual violence (6 percent).

Evidence suggests that IPV has far‐reaching consequences for women. Women with a history of IPV have a higher risk of urinary tract infections, sexually transmitted infections or HIV, pregnancy loss, miscarriage or abortion, and sexual dissatisfaction (Silverman et al. 2008; Dhar et al. 2018; Tadayon et al., 2018). Research has also identified a relationship between IPV and unwanted or mistimed births, unmet need for family planning, and adolescent pregnancy (Stephenson et al. 2008). Altogether, these findings indicate that a woman's exposure to IPV influences her own reproductive control (Chapin, Coleman, and Varner 2011; Munoz, Hellman, and Brunk 2017; Walsh, Slesnick, and Wong 2020).

The association between IPV and lack of reproductive self‐efficacy is well established. Women's perceptions and experiences of reproductive control from husbands or in‐laws may affect their decision to use contraception, particularly in a context in which IPV has occurred (Silverman et al. 2019). In such contexts, women may fear partner responses to contraceptive use. On the other hand, women who experience IPV are also often more prone to covert contraceptive use, so that their partners are not aware and cannot take control or retaliate (Biddlecom and Fapohunda 1998; Silverman et al. 2020). Mittal, Senn, and Carey (2013) found that women in abusive relationships were more likely to report being afraid of asking their partners to use condoms. Wingood and Diclemente (1997) found that women with an abusive partner were more likely to experience physical violence as a direct result of negotiating condom use than women not having an abusive partner. Agrawal et al. (2014) also reported that women who had experienced repeated IPV during the postpartum period had higher fear of contraceptive negotiation. Overall, these findings suggest that IPV can compromise women's use of contraceptives by increasing women's fear to negotiate use with their partners.

Less research has focused on IPV and contraceptive use discontinuation. The literature on the topic of discontinuation has largely focused on demographic and use‐related correlates. More specifically, the existing demographic literature on contraceptive dynamics focuses primarily on documenting contraceptive discontinuation rates, comparison of discontinuation among users of different contraceptive methods, as well as reasons for, and determinants of, discontinuation. NFHS‐4 suggests that 12 percent of women discontinued the use of modern methods of contraception because they wanted to get pregnant (IIPS and ICF 2017). Side effects/health concerns, cost, and availability are the prime reasons for discontinuation or method switching (Ali et al. 2012). Studies have also reported that discontinuation rates vary by type of contraceptive methods (Ali and Cleland 1995; Steele and Curtiss 2003; Bradley et al. 2009; Ali et al. 2012; Modey et al. 2014; Maslyanskaya et al. 2016). These studies indicate that discontinuation was lowest among IUD users and highest among condom and pill users. Recent estimates from FP2020 show that, in India, about 13 percent of IUD users discontinued while in need (Family Planning, 2019). Discontinuation while in need was 30 percent among injectable users and 19 percent among condom and pill users ( Family Planning, 2019). Individual factors commonly associated with discontinuation include fertility desires, parity, education, socioeconomic status, and sometimes age (Grady et al. 1988; Curtis and Blanc 1997; Blanc 2001; Bradley et al. 2009; Ali et al. 2012).

However, there are only a few studies that have examined the experience of IPV as a possible predictor of contraceptive use discontinuation. A study from Bolivia did not find any association between IPV and contraceptive use discontinuation (McCarraher et al. 2006). Using data from selected Demographic and Health Survey (DHS) countries, MacQuarrie, Mallick, and Kishor (2016) found inconsistent evidence of an association between IPV and contraceptive use discontinuation. Where associations existed, they were often of small magnitude or inconsistent in the direction of the association. The association between IPV and contraceptive discontinuation was also found to vary considerably by the form of violence. MacQuarrie, Mallick, and Kishor (2016) found that emotional violence was significantly associated with a greater risk of discontinuation in Egypt, Honduras, and Kenya. However, in the Kyrgyz Republic, emotional violence was significantly associated with a lower risk of discontinuation. Sexual violence was positively associated with discontinuation in Jordan and negatively associated with Tajikistan (MacQuarrie, Mallick, and Kishor 2016). The association between physical violence and discontinuation was marginally significant in Egypt and Honduras. Allsworth et al. (2013) examined the impact of different forms of abuse across the lifespan on contraceptive discontinuation in the USA and found that lifetime experience of sexual violence was significantly associated with discontinuation among users of long‐acting reversible contraceptive methods. No other form of violence was associated with discontinuation in this USA‐based sample. Research from Nigeria examined the effect of any form of IPV on contraceptive discontinuation but did not disaggregate results by type of IPV (Kupoluyi 2020). No study has examined this association in India, despite the fact that 33 percent of women in India who used contraceptives reported discontinuation within 12 months of contraceptive initiation, and most of these do not switch to a different modern contraceptive (Ali. et al. 2014; IIPS and ICF 2017).

Given the lack of such evidence from India and the inconsistent effect of IPV on contraceptive use discontinuation in other countries, the present study examines the association between IPV and contraceptive use discontinuation in India.

## DATA AND METHODS

### Data

Our study uses nationally representative cross‐sectional data from the fourth round of the NFHS conducted in India in 2015–2016. The principal objective of the NFHS‐4 was to provide information on maternal and child health, family planning, other reproductive health indicators as well as domestic violence; sexual behavior; HIV/AIDS knowledge, attitudes, and behavior (IIPS and ICF 2017). A total of 699,686 eligible women aged 15–49 were interviewed, with a response rate of 97 percent (IIPS and ICF 2017).

We used reproductive calendar data collected in NFHS‐4 to examine the association between IPV and contraceptive use discontinuation in India. The reproductive calendar includes a monthly history of key events such as pregnancies, terminations, births, contraceptive use, type of contraceptive methods, and reasons for discontinuation of contraception use for a period of 72 months prior to the survey. The reproductive calendar is the primary data source for estimating contraceptive discontinuation rates and the analysis of other contraceptive dynamics such as contraceptive failure, switching, and postpartum adoption of contraception. Data on discontinuation and other interruptions were obtained through the reproductive calendar canvassed in NFHS‐4.

### Ethics Statement

This research is based on publicly available datasets. These datasets do not contain information that may be used to identify the respondents. These datasets may be downloaded from https://dhsprogram.com/. Hence, our study is exempt from ethical approval.

### Inclusion and Exclusion Criteria

As information on women's experiences of IPV were collected for the 12 months prior to the survey, we define our period of observation as the 12 months preceding the interview. Hence, we excluded contraceptive use windows occurring exclusively prior to the observation period. By doing so, we were able to measure IPV and contraceptive discontinuation in the same 12 months window.

As we intended to examine the association between IPV and contraceptive discontinuation, we included only those women who were using contraception at the start of the calendar (12 months prior to the interview). Hence our measures of IPV refer to the experience of violence coincident with or subsequent to contraceptive use one year prior to the interview.

We included only currently married women in our analysis. Never married women were excluded because they were not asked questions on IPV. We have also excluded previously (but not currently) married women (e.g. divorced, widowed) because they were not necessarily exposed to the risk of IPV in the past year.

Finally, we restricted our analysis to currently married women who were using modern temporary contraceptive methods including the intrauterine device (IUD), oral contraceptive pill, injection, female condom, male condom, emergency contraception, lactational amenorrhea method (LAM), standard days method, and vaginal methods such as diaphragm, foam, and jelly, at the start of the 12‐month observation period (MacQuarrie, Mallick, and Kishor 2016). We excluded those women who indicated that they or their partners were sterilized at the start of the observation period because such women were not at risk of discontinuation. We have also excluded those women who were using traditional methods of contraceptives at the start of the observation period. Subsequently, we classified modern temporary contraceptive methods into two groups: Long‐acting reversible contraceptive methods (LARC) and non‐LARC. LARCs include IUDs, and non‐LARCs include oral contraceptive pills, injections, female condoms, male condoms, emergency contraception, LAM, standard days method, and vaginal methods such as diaphragm, foam, and jelly (MacQuarrie, Mallick, and Kishor 2016).

**FIGURE 1:**
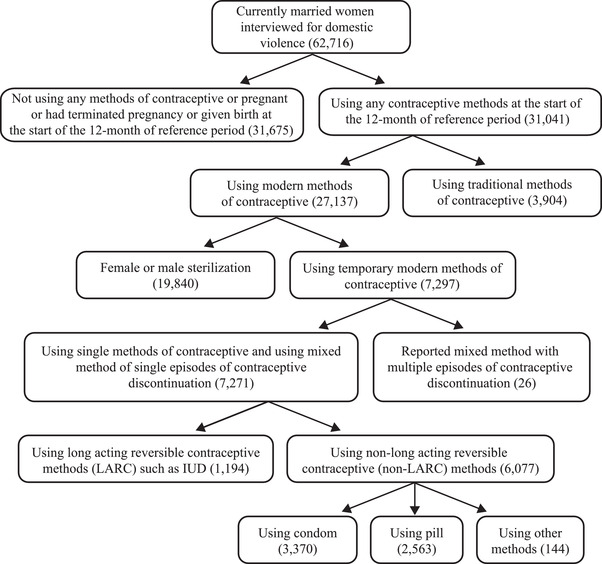
Schematic presentation of the analytical sample, India, 2015–2016

In NFHS‐4, the domestic violence module was canvassed in only a 15‐percent subsample of the original sample—every alternate household in 30 percent of the Primary Sampling Units in the original sample (IIPS and ICF 2017). In addition, only one woman out of all eligible women in the selected 15 percent subsample households was interviewed on the domestic violence module. A total of 62,716 currently married women were interviewed in the domestic violence module. Following exclusions based on analytic sample criteria, the final analytic sample was 7,271 women (Figure 1). Of the 7,271 women who were included in the analysis, 1,194 women were using LARC/IUD and the remaining 6,077 were using non‐LARC methods such as condoms (3,370), pills (2,563), and other methods (144) of contraceptive (Figure 1). All these numbers are unweighted.

### Outcome Variable

**FIGURE 2:**
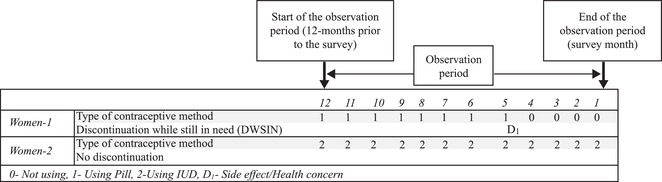
Pictorial representation of contraceptive use discontinuation while still in need

The outcome variable of interest is contraceptive use discontinuation in the past 12 months preceding NFHS‐4. Contraceptive use discontinuation is defined as an interruption in the use of a contraceptive method for one month or longer (MacQuarrie et al., 2016). We created a three‐category variable of contraceptive use discontinuation: did not discontinue (also includes method switching), discontinued while still in need (DWSIN), and discontinued due to no further need (DDNFN). The reasons for discontinuation were asked to all women who stopped using a method of contraception. DWSIN refers to women who want to use contraception but discontinued due to the following reasons: (1) Became pregnant while using, (2) partner disapproved, (3) side effect/health concern, (4) lack of access/too far, (5) wanted more effective method, (6) inconvenient to use, (7) cost too much, (8) other reasons such as lack of sexual satisfaction, created the menstrual problem, gained weight, did not like the method, lack of privacy for users, fatalistic, etc. (MacQuarrie, Mallick, and Kishor 2016). Women who stopped using contraception due to any of the aforementioned reasons during the observation period were coded as 1. For example, if a woman was using pill 12 months prior to the survey and stopped using it after eight months due to side effects was considered as DWSIN (Figure 2). Women who reported discontinuation due to desire for pregnancy, marital dissolution/separation, infrequent sex or husband away, or difficulty to get pregnant/menopausal were included in DDNFN and were coded as 2. Women who did not discontinue or switched to another method without discontinuation were included in did not discontinue, and were coded as 0. Since DWSIN is the key event of interest, we only discuss the results related to DWSIN in the subsequent sections. We show the results related to DDNFN in the Supporting Information.

### Independent Variable

The independent variable of interest is the experience of IPV (categorized as no IPV, physical IPV, emotional IPV, sexual IPV, multiple forms of IPV). NFHS asked a series of questions on women's experience of spousal violence such as physical, emotional, and sexual violence in last 12 months. Physical violence was defined as having ever been pushed, slapped, punched with a fist or hit by something harmful, kicked or dragged, strangled or burned, threatened with knife/gun or other weapon, arm twisted, or hair. Women who reported experience of any of the aforementioned forms of physical violence in last 12 months prior to the survey—either sometimes or often were coded as 1 and the rest were coded as 0. Emotional violence was defined as having ever been humiliated in front of others, threatened to hurt or harm her or someone close to her, and insulted or make her feel bad about herself. Women who reported any form of emotional violence in last 12 months were coded as 1, and the rest were coded as 0. Likewise, sexual violence is defined as having ever been physically forced to have sexual intercourse, physically forced to perform any other sexual activity, and forced with threats or in another way to perform sexual acts. Women who reported any of the aforementioned forms of sexual violence in last 12 months were coded as 1, and the rest were coded as 0. Notably, a woman who reported yes to a form of violence may also have said yes to the other two forms of violence. So, to examine the independent effect of the three forms of violence on contraceptive use discontinuation in India, we created a variable experience of IPV—having five categories—no IPV, physical IPV, emotional IPV, sexual IPV, multiple forms of IPV.

### Control Variables

Sociodemographic variables included age of the women (15–24, 25–34, 35–49), the number of living children (0–1, 2, 3+), women's schooling (no schooling, primary, secondary, higher), marital duration (0–4, 5–9, 10–14, 15+ years), currently working (no, yes), caste (Scheduled Caste/Tribes, non‐Scheduled Caste/Tribes), religion (Hindu, Muslim, others), household wealth quintile (poorest, poor, middle, rich, richest), and place of residence (urban, rural). Additionally, we included the duration of contraceptive use before the start of the observation period (≤12 months, 13–24 months, 25–36 months, 37+ months) and decision‐making on women's healthcare (partner alone, respondent alone, jointly, someone else).

### Statistical Analysis

We first show the descriptive statistics of the type of contraceptive methods used at the start of a calendar (12 months prior to the survey), experience of IPV, and other sociodemographic and residence‐related variables. Then we examine the contraceptive use discontinuation by experience of IPV and sociodemographic and residence‐related characteristics. A series of multivariable multinomial logistic regressions were used to examine the adjusted association of IPV with contraceptive use discontinuation. The multivariable multinomial logistic regression was also used to examine the association between IPV and DWSIN among LARC and non‐LARC method users. Notably, non‐LARC methods, such as condoms and pills, are the most commonly used form of modern contraceptive methods and these are more easily disrupted than less frequently used LARC/IUD methods (IIPS and ICF, 2017). Therefore, we further estimated two separate multivariable multinomial logistic regression models to examine the association of experience of IPV with DWSIN among women who were using condoms and pills at the start of the observation period.

Finally, we performed several sensitivity analyses to examine the robustness of our results. Existing research has shown that past experience of violence (not in last 12 months) may also be associated with contraceptive use discontinuation (Allsworth et al. 2013). To address this potential bias, we disaggregated the reference category of the independent variable into two—never experienced IPV and experienced IPV but not in last 12 months. We then reestimated the regression models with IPV as the independent variable (never experienced IPV, experienced IPV but not in last 12 months, experienced physical IPV in last 12 months, experienced emotional IPV in last 12 months, experienced sexual IPV in last 12 months, experienced multiple forms of IPV in last 12 months) and contraceptive discontinuation as the dependent variable, adjusting for other control variables. Second, since our analysis included only those women who were using temporary modern methods of contraception at the start of the observation period there is a possibility of selection bias in the sense that women's prior experience of IPV may affect the contraceptive choice at the start of the observation period. A few past studies have reported an association between experience of IPV and the type of contraceptive method used (Allsworth et al. 2013; Raj et al. 2015; Tomar et al. 2020). To address this potential selection bias, we tested whether the choice of modern temporary methods of contraception at the start of the observation period varied by experience of IPV in last 12 months against experience of IPV in the past.

We used domestic violence weights in estimations. The details of the sampling weights are given in the NFHS‐4 report (IIPS and ICF 2017). We used svyset and svy suite of commands to account for the complex sampling design of NFHS‐4 in the estimations. All the analysis was done in STATA 15.0.

## RESULTS

**FIGURE 3:**
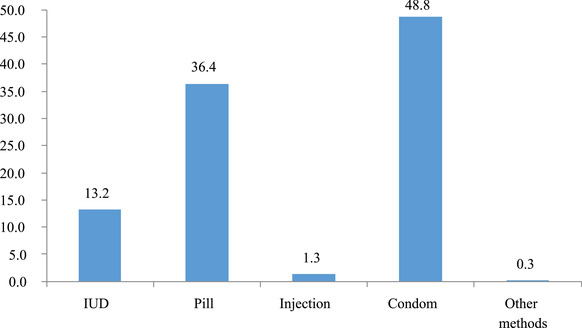
Percentage distribution of currently married women age 15–49 by contraceptive method used at the start of the observation period, India, 2015–2016

About 11 percent of women who were using a contraceptive method at the start of the 12‐month reference period discontinued the use of contraceptives before the end of the period. Of these, about 5.5 percent of women DWSIN and 5.6 percent DDNFN. The type of contraceptive methods used 12 months prior to the survey is shown in Figure 3. A majority of the women were using condoms (48.8 percent), followed by pill (36.4 percent) and LARC/IUD (13.2 percent). Only one percent were using injection and 0.3 percent were using other modern contraceptive methods. Eighty‐seven percent of women were using non‐LARC methods of contraception.

A little more than two‐fifths (21 percent) of women experienced at least one form of IPV (Table 1). Of these, about 10 percent experienced physical IPV, two percent experienced emotional IPV, one percent experienced sexual IPV, and eight percent multiple forms (at least two) of IPV in the past 12 months. More than half of the women had schooling up to the secondary level, were of 25–34 years of age, resided in rural areas, and belonged to rich/richest households (52.1, 56.7, 54.9, 54.0 percent respectively). About 44 percent reported uninterrupted use of contraceptive methods for more than 36 months prior to the start of the observation period.

**TABLE 1:** Sample characteristics, contraceptive discontinuation while still in need (DWSIN) according to experience of IPV, sociodemographic, and residence related characteristics, India, 2015–2016

Background characteristics	*N* (Unweighted)	Sample distribution % (95% CI)	DWSIN % (95% CI)	DWSIN among LARC users % (95% CI)	DWSIN among non‐LARC users % (95% CI)	DWSIN among condom users % (95% CI)	DWSIN among pill users % (95% CI)
IPV in last 12‐months							
No IPV	5776	79.2 (77.8–80.6)	5.2 (4.4–6.1)	5.1 (3.1–8.4)	5.2 (4.4–6.2)	3.8 (3.0–4.8)	7.0 (5.4–8.9)
Physical IPV	717	9 .7 (8.7–10.7)	7.4 (5.1–10.6)	14.4 (7.5–25.8)	6.2 (3.9–9.7)	6.2 (3.6–10.6)	4.4 (2.4–7.8)
Emotional IPV	168	2.3 (1.8–2.9)	9.3 (5.0–16.4)	27.8 (6.4–68.4)	8.3 (4.3–15.6)	16.0 (7.9–29.9)	3.3 (0.8–13.0)
Sexual IPV	88	1.2 (0.8–1.6)	6.2 (1.6–20.8)	1.4 (0.3–6.9)	6.8 (1.7–23.2)	9.4 (1.4–43.9)	5.1 (0.8–27.6)
Multiple forms of IPV	522	7.7 (6.8–8.7)	5.4 (3.7–7.7)	6.0 (2.5–13.5)	5.3 (3.5–7.8)	5.6 (3.4–9.1)	4.2 (2.0–8.7)
Age of women							
15–24	981	15.8 (14.4–17.3)	8.6 (6.5–11.2)	11.6 (5.1–24.0)	8.3 (6.2–11.1)	7.6 (5.2–11.0)	8.0 (4.9–12.9)
25–34	4042	56.7 (54.8–58.6)	5.4 (4.5–6.5)	6.1 (3.6–10.0)	5.3 (4.3–6.4)	4.7 (3.6–6.0)	6.0 (4.4–8.1)
35+	2248	27.5 (25.8–29.2)	4.1 (2.8–5.8)	4.9 (2.6–9.2)	3.9 (2.6–6.0)	2.2 (1.4–3.3)	5.5 (3.4–8.9)
Number of living children							
0–1	2199	36.0 (33.8–38.2)	5.0 (3.9–6.3)	4.5 (2.4–8.1)	5.0 (3.9–6.5)	4.5 (3.2–6.3)	5.3 (3.5–7.9)
2	2997	40.0 (38.0–42.0)	5.4 (4.3–6.7)	7.4 (4.1–13.0)	5.1 (4.0–6.4)	3.7 (2.7–5.1)	6.8 (4.8–9.5)
3+	2075	24.0 (22.6–25.6)	6.6 (5.1–8.4)	7.2 (3.8–13.1)	6.5 (5.0–8.5)	5.5 (4.0–7.6)	6.8 (4.5–10.2)
Duration of use before the observation period							
≤12 months	2097	28.9 (27.4–30.5)	9.8 (8.3–11.6)	7.9 (4.5–13.5)	10.1 (8.4–12.1)	9.3 (7.3–11.8)	10.4 (7.6–14.0)
13–24 months	1198	16.1 (14.8–17.5)	5.9 (4.2–8.2)	8.1 (3.9–16.2)	5.5 (3.7–8.0)	3.4 (2.1–5.6)	8.3 (4.9–13.9)
25–36 months	794	10.6 (9.6–11.7)	5.3 (3.2–8.7)	12.0 (4.6–27.8)	4.1 (2.3–7.1)	2.6 (1.3–5.2)	6.0 (2.7–12.9)
37+ months	3182	44.4 (42.5–46.3)	2.7 (1.8–3.8)	2.8 (1.5–5.3)	2.6 (1.8–3.9)	1.6 (1.1–2.5)	3.4 (2.1–5.4)
Women's schooling							
No schooling	1388	16.2 (15.1–17.5)	5.5 (4.1–7.4)	3.9 (1.1–12.6)	5.7 (4.1–7.7)	5.1 (3.4–7.5)	6.2 (3.8–10.0)
Primary	914	12.7 (11.5–14.1)	5.9 (4.0–8.6)	7.3 (2.6–18.8)	5.7 (3.8–8.6)	6.7 (3.9–11.2)	3.8 (2.0–7.1)
Secondary	3778	52.1 (50.1–54.0)	6.1 (5.1–7.3)	7.6 (4.7–12.0)	5.9 (4.8–7.1)	4.4 (3.4–5.7)	7.3 (5.4–9.9)
Higher	1191	18.9 (17.4–20.6)	3.8 (2.6–5.4)	4.4 (2.0–9.2)	3.6 (2.4–5.4)	3.2 (1.9–5.3)	5.4 (2.9–10.0)
Marital duration							
0–4 years	1089	15.5(14.2–16.9)	8.3 (6.3–10.8)	6.3 (2.8–13.3)	8.6 (6.4–11.3)	6.8 (4.6–9.9)	10.3 (6.4–16.2)
5–9 years	2047	29.4(27.5–31.3)	5.8 (4.6–7.3)	6.8 (3.7–12.1)	5.7 (4.4–7.3)	5.1 (3.6–7.2)	6.2 (4.2–9.1)
10–14 years	1911	25.7 (24.2–27.3)	5.3 (3.9–7.1)	7.1 (3.1–15.7)	5.0 (3.6–6.9)	4.1 (2.7–6.0)	6.1 (3.7–9.7)
15+ years	2224	29.4 (27.8–31.1)	4.0 (2.8–5.7)	5.1 (2.7–9.5)	3.8 (2.6–5.7)	2.5 (1.7–3.7)	4.7 (3.0–7.5)
Decision on women's health care							
Respondent alone	692	11.2 (10.0–12.4)	6.9 (5.4–8.9)	5.0 (2.2–11.3)	7.2 (5.5–9.3)	6.5 (4.5–9.2)	7.8 (5.2–11.5)
Partner alone	1297	17.3 (16.1–18.7)	6.4 (3.9–10.2)	10.0 (3.5–25.1)	5.6 (3.2–9.8)	5.9 (3.1–10.7)	3.4 (1.0–10.9)
Jointly	5141	69.6 (67.9–71.2)	4.9 (4.2–5.8)	5.0 (3.3–7.6)	4.9 (4.1–5.8)	3.7 (2.9–4.7)	6.3 (4.8–8.1)
Someone else	141	1.9 (1.6–2.4)	10.2 (5.3–18.7)	32.7 (11.2–65.3)	6.4 (3.1–12.8)	5.0 (2.0–11.9)	9.4 (2.3–31.6)
Currently working							
No	5824	80.4 (78.7–82.0)	6.0 (5.1–6.9)	7.1 (4.7–10.6)	5.8 (4.9–6.8)	4.7 (3.8–5.7)	6.8 (5.3–8.5)
Yes	1447	19.6 (18.0–21.3)	3.7 (2.7–5.1)	4.1 (1.9–8.7)	3.6 (2.6–5.1)	3.4 (2.1–5.3)	4.0 (2.4–6.7)
Wealth quintile							
Poorest	761	11.0 (9.8–12.3)	6.2 (4.1–9.2)	7.8 (2.6–21.2)	6.0 (3.9–9.3)	5.7 (2.9–11.0)	6.3 (3.6–10.7)
Poorer	1337	17.7 (16.2–19.3)	6.0 (4.4–8.2)	3.4 (1.4–8.0)	6.2 (4.5–8.6)	7.2 (4.6–10.9)	4.8 (3.0–7.5)
Middle	1430	17.2 (15.8–18.7)	5.6 (4.1–7.5)	3.2 (0.8–12.4)	5.8 (4.2–7.9)	4.8 (3.2–7.1)	6.8 (4.2–10.7)
Rich	1524	20.5 (19.1–22.1)	6.8 (5.3–8.9)	12.6 (7.8–19.6)	5.9 (4.3–8.0)	4.3 (2.9–6.3)	8.8 (5.3–14.3)
Richest	2219	33.5 (31.5–35.5)	4.3 (3.3–5.5)	4.6 (2.2–9.5)	4.2 (3.2–5.5)	3.5 (2.5–5.0)	5.9 (3.7–9.4)
Religion							
Hindu	4786	72.3 (69.6–74.8)	5.2 (4.4–6.0)	6.4 (4.1–9.8)	5.0 (4.2–5.9)	4.2 (3.3–5.2)	5.9 (4.6–7.6)
Muslim	1412	20.8 (18.4–23.5)	7.0 (5.2–9.3)	7.7 (3.2–17.3)	6.9 (5.1–9.4)	5.6 (3.7–8.4)	7.0 (4.7–10.4)
Others	1073	6.9 (6.0–8.0)	4.9 (2.9–8.3)	4.6 (1.8–11.2)	5.0 (2.7–9.3)	3.7 (2.0–6.4)	7.6 (2.4–21.5)
Caste							
SC/ST	2300	24.0 (22.3–25.9)	5.6 (4.3–7.1)	5.9 (3.2–10.6)	5.5 (4.2–7.2)	5.5 (3.8–7.9)	5.2 (3.5–7.8)
Non SC/ST	4971	76.0 (74.1–77.7)	5.5 (4.7–6.5)	6.4 (4.1–9.9)	5.4 (4.5–6.4)	4.1 (3.3–5.1)	6.7 (5.2–8.6)
Place of residence							
Urban	2756	45.1 (43.3–47.0)	4.7 (3.8–5.8)	5.9 (3.2–10.7)	4.5 (3.5–5.7)	3.9 (2.9–5.3)	5.6 (3.6–8.6)
Rural	4515	54.9 (53.0–56.7)	6.2 (5.2–7.4)	6.8 (4.6–10.1)	6.1 (5.1–7.4)	5.0 (3.9–6.3)	6.6 (5.1–8.4)
Total	7271	100.0	5.5 (4.8–6.3)	6.3 (4.4–9.1)	5.4 (4.7–6.3)	4.4 (3.6–5.3)	6.3 (5.0–7.8)

NOTES: Percentage discontinuation due to no further need (DDNFN) according to experience of IPV, sociodemographic, and residence‐related characteristics are shown in online supplementary Table T1.

Abbreviations: DWSIN: , discontinuation while still in need; IUD, intrauterine device; IPV, intimate partner violence; LARC, long‐acting reversible contraceptive.

DWSIN by experience of IPV and other sociodemographic and residence‐related characteristics are also shown in Table 1. Six percent of women DWSIN within the 12 months observation period. DWSIN was highest among those who experienced emotional violence (9.3 percent) followed by those who experienced physical violence (7.4 percent) and those who experienced sexual violence (6.2 percent). DWSIN was about 5.4 percent among those who experienced multiple forms of violence. DWSIN was lowest among women who did not experience any form of partner violence (5.2 percent) in the last 12 months. Further results show that experience of IPV was associated with higher discontinuation among those women who were using LARC/IUD and condoms at the start of the observation period. Interestingly, experience of IPV was not associated with higher discontinuation among women who were using the pill at the start of the observation period.

Results of multivariable multinomial logistic regression analyses examining the association of IPV with DWSIN are shown in Table 2. No evidence of a difference in the risk of DWSIN was found between women who experienced physical, emotional, or sexual violence and women who did not experience any violence in the last 12‐months. Experience of multiple forms of IPV was also not associated with a higher risk of DWSIN.

**TABLE 2:** Results of multivariable multinomial logistic regression analysis showing adjusted relative risk ratio (RRR) for examining the association between experience of IPV and contraceptive use discontinuation, India, 2015–2016

	DWSIN
Intimate partner violence in last 12‐months	RRR (95% CI)
	
No IPV	1.00
Physical IPV	1.25 (0.82–1.90)
Emotional IPV	1.59 (0.77–3.28)
Sexual IPV	1.10 (0.27–4.39)
Multiple forms of IPV	0.88 (0.56–1.39)

NOTES: Models are adjusted for age of the women, the number of living children, duration of contraceptive use before the start of the observation period, women's schooling, marital duration, decision on women's healthcare, currently working, caste, religion, wealth quintile, and place of residence. RRR showing association between experience of IPV and discontinuation due to no further need (DDNFN) are shown in online supplementary Table T2

Abbreviations: DWSIN, discontinuation while still in need; IPV, intimate partner violence; RRR, relative risk ratio.

Adjusted relative risk ratios (RRR) for examining the association between experience of IPV and DWSIN among users of LARC/IUD and non‐LARC methods are shown in Table 3. Experience of physical violence was significantly associated with DWSIN among women using LARC/IUD methods of contraception (RRR: 3.73, 95 percent CI [1.55–8.95]). No other form of IPV was associated with DWSIN among those using LARC and non‐LARC methods.

**TABLE 3:** Results of multivariable multinomial logistic regression analysis showing adjusted relative risk ratio for examining the association between experience of IPV and contraceptive use discontinuation among LARC non‐LARC, condom and pill users, India, 2015–2016

	DWSIN among LARC/IUD users	DWSIN among Non‐LARC users	DWSIN among Condom users	DWSIN among Pill users
IPV in last 12‐months	RRR (95% CI)	RRR (95% CI)	RRR (95% CI)	RRR (95% CI)
No IPV	1.00	1.00	1.00	1.00
Physical IPV	3.73 (1.55–8.95)	1.03 (0.62–1.70)	1.30 (0.67–2.55)	0.56 (0.28–1.13)
Emotional IPV	5.76 (0.88–37.91)	1.37 (0.62–3.02)	4.16 (1.59–10.90)	0.39 (0.09–1.81)
Sexual IPV	0.19 (0.02–1.82)	1.23 (0.30–5.09)	2.13 (0.28–16.14)	0.80 (0.11–5.71)
Multiple forms of IPV	1.29 (0.36–4.54)	0.84 (0.52–1.38)	1.13 (0.56–2.25)	0.51 (0.22–1.18)

NOTES: Models are adjusted for age of the women, number of living children, duration of contraceptive use before the start of the observation period, women's schooling, marital duration, decision on women's healthcare, currently working, caste, religion, wealth quintile, and place of residence. RRR showing the association between experience of IPV and discontinuation due to no further need (DDNFN) among LARC/IUD, non‐LARC, condom, and pill users are shown in online supplementary Tables T3, T4, T5, and T6, respectively.

Abbreviations: IPV, intimate partner violence; IUD, intrauterine device; LARC, long‐acting reversible contraceptive; RRR, relative risk ratio.

Adjusted RRRs for the association of IPV with DWSIN among women using condoms or pills at the start of the observation period are also shown in Table 3. Among the condom users, the risk of DWSIN was significantly higher among women who experienced emotional violence (RRR: 4.16, 95 percent CI [1.59–10.90]) compared with women who did not experience any form of violence in last 12 months. No associations were significant among the pill users.

### Sensitivity Analysis

We performed several sensitivity analyses to check the robustness of the associations. The association between IPV and DWSIN remained unchanged when we used an alternative six‐level IPV measure (Table 4). Findings of the second sensitivity analysis clearly show that the choice of contraceptive methods, such as LARC/IUD, non‐LARC, condom or pill, does not differ substantially by experience of IPV in the last 12 months against experience of IPV in the past (but not in last 12 months) (Table 5).

**TABLE 4:** Results of multivariable multinomial logistic regression for examining the association between timing of IPV and contraceptive use DWSIN, among LARC, non‐LARC, condom and pill users, India, 2015–2016

	DWSIN	DWSIN among LARC/IUD users	DWSIN among Non‐LARC users	DWSIN among Condom users	DWSIN among Pill users
Experience of IPV	RRR (95% CI)	RRR (95% CI)	RRR (95% CI)	RRR (95% CI)	RRR (95% CI)
Never experienced IPV	1.00	1.00	1.00	1.00	1.00
Experienced IPV but not in last 12 months	1.18 (0.64–2.15)	0.80 (0.11–5.83)	1.21 (0.64–2.29)	1.49 (0.67–3.34)	1.08 (0.41–2.90)
Experienced physical IPV in last 12 months	1.27 (0.81–1.99)	3.69 (1.52–8.95)	1.05 (0.61–1.81)	1.36 (0.69–2.68)	0.57 (0.28–1.15)
Experienced emotional IPV in last 12 months	1.62 (0.78–3.35)	5.81 (0.89–37.98)	1.40 (0.63–3.10)	4.30 (1.64–11.28)	0.40 (0.09–1.83)
Experienced sexual IPV in last 12 months	1.11 (0.28–4.47)	0.19 (0.02–1.80)	1.26 (0.30–5.21)	2.21 (0.29–16.75)	0.80 (0.11–5.79)
Experienced multiple forms of IPV in last 12 months	0.90 (0.57–1.41)	1.28 (0.36–4.52)	0.86 (0.53–1.41)	1.17 (0.59–2.35)	0.52 (0.22–1.19)

NOTES: Models are adjusted for‐ age of the women, number of living children, duration of contraceptive use before the start of the observation period, women's schooling, marital duration, decision on women's healthcare, currently working, caste, religion, wealth quintile, and place of residence.

Abbreviations: IPV, DWSIN, discontinuation while still in need; intimate partner violence; LARC: long‐acting reversible contraceptive; RRR, relative risk ratio.

**TABLE 5:** Choice of contraceptive methods (LARC, non‐LARC; condom and pill) at the start of the observation period by the timing of IPV in India 2015–2016

	LARC and Non‐LARC		Non‐LARC	
Timing of IPV	LARC/IUD (%)	Non‐LARC (%)	Condom(%)	Pill (%)
Experienced IPV but not in last 12 months	8.0	92.0	46.5	53.5
Experienced IPV in last 12 months	13.0	87.0	50.2	49.8

NOTES: IPV: Intimate Partner Violence, LARC: Long‐acting reversible contraceptive.

## DISCUSSION

Using reproductive calendar data from the fourth round of NFHS (2015–2016), the present study examined the association between experience of IPV and contraceptive use discontinuation in India among currently married women using nonpermanent modern contraceptive methods. Condoms and pills were the most common forms of contraceptive method (48.4 and 36.4 percent), followed by IUD (13.2 percent), injection (1.3 percent), and other modern methods (0.3 percent). Findings reveal that over the 12‐month observation period, about six percent of sampled women reported DWSIN. Our overall model did not indicate significant associations between IPV and DWSIN. However, among those reporting LARC (IUD) at the start of the observation period, regression results indicate that women who experienced physical IPV were over three times as likely as women who did not experience any IPV to report DWSIN. This finding is consistent with that of prior research from the United States, which documented associations between violence and LARC use, as well as with non‐LARC methods (Allsworth et al. 2013). Given prior research from India indicating that IUD users were least likely of all contraceptive users by type to report discontinuation (IIPS and ICF 2017) combined with current national efforts to expand IUD use in the country, these findings offer important implications for the field. There is a need for assessing IPV and contraceptive choice as part of contraceptive counseling and support, an approach used and proven effective in other country contexts (Tancredi et al. 2015; Miller et al. 2016).

Non‐LARC methods include condoms, pills, injections, and other modern methods of contraception, and these are more easily discontinued than the LARC/IUD method. Prior research from India, Bangladesh, and the United States have reported lower likelihood of condoms and greater likelihood of oral contraceptive pill use among abused women (Silverman et al. 2007, 2011; Raj et al. 2015). Therefore, knowing the widespread use of condoms and pills relative to LARC in India, we further examined the association between experience of IPV and DWSIN among condom and pill users. Among condom users, women who experienced emotional violence were 4.16 times as likely as women who did not experience any violence to DWSIN. No such associations were observed among the pill users. While consistent condom use requires a male partner's approval and participation, oral contraceptive pills do not (Reed et al. 2016). This may be the reason why condom use discontinuation is higher among the women using condoms and who experience emotional violence compared with women using condoms but not experiencing violence. Previous studies from India have also reported that women who had abusive male partners were more likely to use contraceptives without informing their partner in order to redress the reproductive control (Reed et al. 2016; McDougal et al. 2020). MacQuarrie, Mallice, and Kishor (2016) also examined the impact of IPV on contraceptive use discontinuation using DHS data from several countries of Asia, the Middle East, and Africa and found no evidence on the effect of IPV on contraceptive use discontinuation among LARC or non‐LARC users. They did not, however, examine the independent effect of IPV on discontinuation for specific methods of contraception such as condoms, pills, and IUDs. To the best of our knowledge, this is the first study from India which has examined the independent effect of each of three forms of IPV on the contraceptive use discontinuation using reproductive calendar data.

This study has some limitations. First, we could not establish the exact timing of occurrence of IPV, so we cannot say with confidence if the IPV preceded women's discontinued use of contraceptive methods. However, we were able to co‐locate IPV and contraceptive use discontinuation in the same 12 months observation period (MacQuarrie, Mallice, and Kishor 2016), which other cross‐sectional studies could not do. Additionally, while these analyses are not definitively causal, our sensitivity analyses helped address several potential biases, advancing our results along the causal pathway. Second, experience of IPV was self‐reported by women and thus is subject to recall and social desirability bias. Third, we could not examine the separate effects of IPV among those who were using injections and other modern temporary methods of family planning at the start of the observation period, as the use of these methods in India is very limited. Finally, our conclusions are necessarily limited to the women represented in our analytic sample, namely currently married women using contraception one year prior to an interview, and we are unable to draw conclusions about other groups of women.

The findings of our study provide compelling evidence on the association between experience of IPV and contraceptive use discontinuation in India, particularly in the case of LARC and condom use. While 11 percent of women who experienced physical violence discontinued use of IUD, eight percent of women who experienced emotional violence discontinued use of condoms. Extrapolating these figures to the population of India suggest that about 78,000 women aged 15–49 are likely to discontinue the use of IUD annually in India owing to episode(s) of physical violence, and about 100,000 women aged 15–49 are likely to discontinue use of condoms in India annually owing to episode(s) of emotional violence. These high figures are important given the Government of India's FP2020 commitments. At the 2012 summit, the Government of India committed to spend USD 2.0 billion by 2020 for its family planning program. In July 2017, the Government of India renewed its commitment to invest USD 3.0 billion by 2020 ( Family Planning, 2019). Additionally, the Government of India committed to add 15.5 million additional users by 2020 (Ministry of Statistics and Programme Implementation [MoSPI] 2018). Such high figures of discontinuation will have serious bearing on India's commitment to FP2020. Moreover, the *Statistical Yearbook India 2018* suggests that about six million IUD insertions happened in 2015–2016. Likewise, there were about 11 million condom users in 2015–2016 ( Family Planning, 2019). Given the above discontinuation figures, approximately 60,000 IUD users and 200,000 condom users are likely to discontinue using these methods due to experience of IPV annually. Given these big numbers, there is a clear need to understand the women's experience of IPV as a part of a broader strategy to provide high‐quality family planning services to all women while considering individual circumstances and reproductive aspiration to support the uninterrupted use of contraception in India.

## CONFLICT OF INTEREST

The authors declare no conflict of interest.

## ETHICS APPROVAL STATEMENT

This research is based on publicly available datasets. These datasets do not contain information that may be used to identify the respondents. These datasets may be downloaded from https://dhsprogram.com/. Hence, no ethical approval was required for this study.

## Supporting information

Table T1. Percentage of contraceptive discontinuation due to no further need (DDNFN) according to experience of IPV, socio‐demographic, and residence related characteristics, India, 2015‐16
Table T2. Results of multinomial logistic regression analysis showing adjusted relative risk ratio (RRR) for examining the association between experience of IPV and contraceptive use discontinuation, India, 2015‐16
Table T3. Results of multinomial logistic regression analysis showing adjusted relative risk ratio (RRR) for examining the association between experience of IPV and contraceptive use discontinuation among LARC/IUD users, India, 2015‐16
Table T4. Results of multinomial logistic regression analysis showing adjusted relative risk ratio (RRR) for examining the association between experience of IPV and contraceptive use discontinuation among non‐LARC users, India, 2015‐16
Table T5. Results of multinomial logistic regression analysis showing adjusted relative risk ratio (RRR) for examining the association between experience of IPV and contraceptive use discontinuation among condom users, India, 2015‐16
Table T6. Results of multinomial logistic regression analysis showing adjusted relative risk ratio (RRR) for examining the association between experience of IPV and contraceptive use discontinuation among pill users, India, 2015‐16

## Data Availability

Our analysis is based on NFHS‐4. The NFHS‐4 is a publicly available dataset with no individual identifiers in place. The NFHS‐4 dataset may be freely accessed from the DHS Program website: https://dhsprogram.com.
