# Supplementary material for: Association between Intimate Partner Violence and Contraceptive Use Discontinuation in India
Source: Stud Fam Plann. 2022 Jan 15;53(1):5–21. doi: 10.1111/sifp.12184 (PMC8957512; doi:10.1111/sifp.12184)
Supplement: Supplementary file 1 — Table T1. Percentage of contraceptive discontinuation due to no further need (DDNFN) according to experience of IPV, socio‐demographic, and residence related characteristics, India, 2015‐16 Table T2. Results of multinomial logistic regression analysis showing adjusted relative risk ratio (RRR) for examining the association between experience of IPV and contraceptive use discontinuation, India, 2015‐16 Table T3. Results of multinomial logistic regression analysis showing adjusted relative risk ratio (RRR) for examining the association between experience of IPV and contraceptive use discontinuation among LARC/IUD users, India, 2015‐16 Table T4. Results of multinomial logistic regression analysis showing adjusted relative risk ratio (RRR) for examining the association between experience of IPV and contraceptive use discontinuation among non‐LARC users, India, 2015‐16 Table T5. Results of multinomial logistic regression analysis showing adjusted relative risk ratio (RRR) for examining the association between experience of IPV and contraceptive use discontinuation among condom users, India, 2015‐16 Table T6. Results of multinomial logistic regression analysis showing adjusted relative risk ratio (RRR) for examining the association between experience of IPV and contraceptive use discontinuation among pill users, India, 2015‐16 [file SIFP-53-5-s001.docx]

**Online Only Supplementary Material**

Association between intimate partner violence and contraceptive use discontinuation in India

| **Table T1. Percentage of contraceptive discontinuation due to no further need (DDNFN) according to experience of IPV, socio-demographic, and residence related characteristics, India, 2015-16** | |
| --- | --- |
| **Background characteristics** | **DDNFN** |
|  | **% (95% CI)** |
| **IPV in last 12-months** |  |
| No IPV | 5.7 (4.8-6.8) |
| Physical IPV | 3.9 (2.6-5.9) |
| Emotional IPV | 7.0 (2.6-17.8) |
| Sexual IPV | 6.6 (1.3-27.2) |
| Multiple forms of IPV | 5.5 (3.6-8.2) |
| **Age of women** |  |
| 15-24 | 9.8 (7.5-12.6) |
| 25-34 | 6.1 (4.9-7.5) |
| 35+ | 2.1 (1.4-3.3) |
| **Number of living children** |  |
| 0-1 | 9.9 (8.1-12.1) |
| 2 | 3.2 (2.4-4.3) |
| 3+ | 2.9 (2.0-4.2) |
| **Duration of use before the observation period** |  |
| 1-12 months | 8.7 (6.8-11.0) |
| 13-24 months | 7.7 (5.7-10.3) |
| 25-36 months | 5.2 (3.6-7.5) |
| 37+ months | 2.8 (1.9-4.2) |
| **Women's schooling** |  |
| No schooling | 4.3 (3.0-6.1) |
| Primary | 4.7 (3.1-7.0) |
| Secondary | 5.7 (4.6-7.0) |
| Higher | 6.8 (4.7-9.8) |
| **Marital duration (in years)** |  |
| 0-4 years | 13.1 (10.2-16.7) |
| 5-9 years | 8.2 (6.3-10.6) |
| 10-14 years | 2.6 (1.9-3.6) |
| 15+ years | 1.6 (.9-2.7) |
| **Decision on women’s health care** |  |
| Respondent alone | 6.1 (4.6-8.0) |
| Partner alone | 5.8 (3.4-9.8) |
| Jointly | 5.4 (4.4-6.6) |
| Someone else | 5.1 (2.4-10.5) |
| **Currently working** |  |
| No | 5.9 (4.9-7.0) |
| Yes | 4.4 (2.9-6.5) |
| **Wealth quintile** |  |
| Poorest | 6.1 (4.1-8.8) |
| Poorer | 4.3 (2.9-6.4) |
| Middle | 5.2 (3.5-7.6) |
| Rich | 7.1 (5.1-9.9) |
| Richest | 5.3 (4.0-6.9) |
| **Religion** |  |
| Hindu | 5.1 (4.2-6.2) |
| Muslim | 6.6 (4.7-9.1) |
| Others | 7.7 (5.4-10.9) |
| **Caste** |  |
| Scheduled cases/tribes | 5.5 (4.4-7.0) |
| Non Scheduled cases/tribes | 5.6 (4.6-6.8) |
| **Place of residence** |  |
| Urban | **5.3 (4.0-7.0)** |
| Rural | 5.8 (4.8-6.9) |
| **Total** | **5.6 (4.7-6.5)** |

| **Table T2. Results of multinomial logistic regression analysis showing adjusted relative risk ratio (RRR) for examining the association between experience of IPV and contraceptive use discontinuation, India, 2015-16** | | |
| --- | --- | --- |
|  | **DWSIN** | **DDNFN** |
|  | **RRR (95% CI)** | **RRR (95% CI)** |
| **IPV in last 12-months** |  |  |
| No IPV | 1.00 | 1.00 |
| Physical IPV | 1.25 (0.82-1.90) | 0.72 (0.44-1.17) |
| Emotional IPV | 1.59 (0.77-3.28) | 1.44 (0.51-4.04) |
| Sexual IPV | 1.10 (0.27-4.39) | 1.20 (0.25-5.83) |
| Multiple forms of IPV | 0.88 (0.56-1.39) | 0.97 (0.58-1.63) |
| **Age of women** |  |  |
| 15-24 | 1.00 | 1.00 |
| 25-34 | 0.87 (0.58-1.31) | 1.29 (0.81-2.04) |
| 35+ | 1.02 (0.58-1.77) | 1.27 (0.68-2.37) |
| **Number of living children** |  |  |
| 0-1 | 1.00 | 1.00 |
| 2 | 1.50 (0.96-2.32) | 0.48 (0.32-0.73) |
| 3+ | 1.90 (1.11-3.26) | 0.63 (0.36-1.10) |
| **Duration of use before the observation period** |  |  |
| 1-12 months | 1.00 | 1.00 |
| 13-24 months | 0.58 (0.39-0.88) | 0.96 (0.63-1.48) |
| 25-36 months | 0.52 (0.29-0.93) | 0.77 (0.43-1.37) |
| 37+ months | 0.27 (0.17-0.41) | 0.65 (0.34-1.23) |
| **Women's schooling** |  |  |
| No schooling | 1.00 | 1.00 |
| Primary | 1.14 (0.65-2.02) | 0.97 (0.55-1.72) |
| Secondary | 1.23 (0.78-1.94) | 0.82 (0.52-1.28) |
| Higher | 0.86 (0.45-1.64) | 0.79 (0.40-1.56) |
| **Marital duration (in years)** |  |  |
| 0-4 years | 1.00 | 1.00 |
| 5-9 years | 0.71 (0.44-1.15) | 0.78 (0.43-1.42) |
| 10-14 years | 0.68 (0.38-1.20) | 0.27 (0.13-0.58) |
| 15+ years | 0.57 (0.31-1.05) | 0.17 (0.06-0.48) |
| **Decision on women’s health care** |  |  |
| Respondent alone | 1.00 | 1.00 |
| Partner alone | 1.15 (0.67-1.99) | 1.11 (0.58-2.11) |
| Jointly | 0.79 (0.56-1.10) | 0.86 (0.58-1.26) |
| Someone else | 1.25 (0.56-2.80) | 0.65 (0.27-1.59) |
| **Currently working** |  |  |
| No | 1.00 | 1.00 |
| Yes | 0.68 (0.46-1.01) | 0.81 (0.50-1.29) |
| **Wealth quintile** |  |  |
| Poorest | 1.00 | 1.00 |
| Poorer | 0.95 (0.53-1.70) | 0.60 (0.33-1.10) |
| Middle | 1.01 (0.55-1.83) | 0.78 (0.45-1.35) |
| Rich | 1.42 (0.79-2.57) | 1.12 (0.61-2.03) |
| Richest | 1.13 (0.59-2.16) | 0.83 (0.44-1.57) |
| **Religion** |  |  |
| Hindu | 1.00 | 1.00 |
| Muslim | 1.23 (0.79-1.90) | 1.51 (0.95-2.41) |
| Others | 0.90 (0.49-1.68) | 1.65 (1.04-2.63) |
| **Caste** |  |  |
| Scheduled cases/tribes | 1.00 | 1.00 |
| Non Scheduled cases/tribes | 1.03 (0.73-1.46) | 1.03 (0.70-1.52) |
| **Place of residence** |  |  |
| Urban | 1.00 | 1.00 |
| Rural | 1.34 (0.96-1.87) | 1.31 (0.90-1.90) |

| **Table T3. Results of multinomial logistic regression analysis showing adjusted relative risk ratio (RRR) for examining the association between experience of IPV and contraceptive use discontinuation among LARC/IUD users, India, 2015-16** | | |
| --- | --- | --- |
|  | **LARC/IUD** | |
|  | **DWSIN** | **DDNFN** |
|  | **RRR (95% CI)** | **RRR (95% CI)** |
| **IPV in last 12-months** |  |  |
| No IPV | 1.00 | 1.00 |
| Physical IPV | 3.73 (1.55-8.95) | 0.15 (0.02-1.09) |
| Emotional IPV | 5.76 (0.88-37.91) | 6.21 (1.05-36.88) |
| Sexual IPV | 0.19 (0.02-1.82) | * |
| Multiple forms of IPV | 1.29 (0.36-4.54) | 0.19 (0.02-2.23) |
| **Age of women** |  |  |
| 15-24 | 1.00 | 1.00 |
| 25-34 | 0.88 (0.28-2.77) | 21.78 (4.46-106.32) |
| 35+ | 1.06 (0.28-4.05) | 8.99 (1.20-67.27) |
| **Number of living children** |  |  |
| 0-1 | 1.00 | 1.00 |
| 2 | 1.12 (0.35-3.59) | 0.62 (0.22-1.73) |
| 3+ | 1.12 (0.25-5.02) | 0.36 (0.07-1.91) |
| **Duration of use before the observation period** |  |  |
| 1-12 months | 1.00 | 1.00 |
| 13-24 months | 1.21 (0.46-3.14) | 1.91 (0.56-6.46) |
| 25-36 months | 1.57 (0.49-5.03) | 3.91 (0.88-17.36) |
| 37+ months | 0.41 (0.11-1.53) | 2.93 (0.47-18.47) |
| **Women's schooling** |  |  |
| No schooling | 1.00 | 1.00 |
| Primary | 1.77 (0.23-13.68) | 4.46 (0.61-32.36) |
| Secondary | 2.60 (0.50-13.56) | 0.31 (0.05-1.79) |
| Higher | 1.83 (0.27-12.50) | 0.27 (0.04-1.70) |
| **Marital duration (in years)** |  |  |
| 0-4 years | 1.00 | 1.00 |
| 5-9 years | 0.81 (0.21-3.17) | 0.86 (0.28-2.66) |
| 10-14 years | 1.53 (0.28-8.37) | 0.07 (0.01-0.50) |
| 15+ years | 0.87 (0.10-7.60) | 0.04 (0.00-0.57) |
| **Decision on women’s health care** |  |  |
| Respondent alone | 1.00 | 1.00 |
| Partner alone | 2.30 (0.57-9.29) | 1.66 (0.27-10.08) |
| Jointly | 1.07 (0.39-2.94) | 0.95 (0.25-3.62) |
| Someone else | 9.81 (1.55-62.07) | 1.38 (0.18-10.83) |
| **Currently working** |  |  |
| No | 1.00 | 1.00 |
| Yes | 0.54 (0.20-1.45) | 0.38 (0.12-1.20) |
| **Wealth quintile** |  |  |
| Poorest | 1.00 | 1.00 |
| Poorer | 0.39 (0.08-1.90) | 0.21 (0.01-3.42) |
| Middle | 0.32 (0.05-2.31) | 0.46 (0.02-9.32) |
| Rich | 1.71 (0.40-7.30) | 1.90 (0.14-25.23) |
| Richest | 0.75 (0.16-3.38) | 1.60 (0.09-29.94) |
| **Religion** |  |  |
| Hindu | 1.00 | 1.00 |
| Muslim | 1.64 (0.64-4.19) | 2.07 (0.62-6.92) |
| Others | 0.62 (0.20-1.91) | 0.95 (0.21-4.40) |
| **Caste** |  |  |
| Scheduled cases/tribes | 1.00 | 1.00 |
| Non ccheduled cases/tribes | 1.02 (0.41-2.56) | 0.65 (0.20-2.07) |
| **Place of residence** |  |  |
| Urban | 1.00 | 1.00 |
| Rural | 1.67 (0.78-3.59) | 0.50 (0.16-1.58) |
| Note. * Insufficient event of DDNFN occurred among those who reported sexual violence in last 12-months. | | |

| **Table T4. Results of multinomial logistic regression analysis showing adjusted relative risk ratio (RRR) for examining the association between experience of IPV and contraceptive use discontinuation among non-LARC users, India, 2015-16** | | |
| --- | --- | --- |
|  | **Non-LARC** | |
|  | **DWSIN** | **DDNFN** |
|  | **RRR (95% CI)** | **RRR (95% CI)** |
| **IPV in last 12-months** |  |  |
| No IPV | 1.00 | 1.00 |
| Physical IPV | 1.03 (0.62-1.70) | 0.80 (0.48-1.31) |
| Emotional IPV | 1.37 (0.62-3.02) | 1.37 (0.46-4.01) |
| Sexual IPV | 1.23 (0.30-5.09) | 1.28 (0.28-5.83) |
| Multiple forms of IPV | 0.84 (0.52-1.38) | 1.09 (0.64-1.85) |
| **Age of women** |  |  |
| 15-24 | 1.00 | 1.00 |
| 25-34 | 0.93 (0.60-1.44) | 1.18 (0.74-1.90) |
| 35+ | 1.09 (0.60-2.01) | 1.22 (0.64-2.36) |
| **Number of living children** |  |  |
| 0-1 | 1.00 | 1.00 |
| 2 | 1.44 (0.89-2.32) | 0.50 (0.30-0.82) |
| 3 | 1.92 (1.06-3.48) | 0.69 (0.37-1.28) |
| **Duration of use before the observation period** |  |  |
| 1-12 months | 1.00 | 1.00 |
| 13-24 months | 0.52 (0.33-0.83) | 0.96 (0.60-1.54) |
| 25-36 months | 0.39 (0.20-0.75) | 0.67 (0.35-1.26) |
| 37+ months | 0.26 (0.17-0.41) | 0.62 (0.31-1.26) |
| **Women's schooling** |  |  |
| No schooling | 1.00 | 1.00 |
| Primary | 1.12 (0.61-2.05) | 0.86 (0.48-1.54) |
| Secondary | 1.16 (0.71-1.90) | 0.88 (0.55-1.40) |
| Higher | 0.82 (0.41-1.65) | 0.93 (0.46-1.88) |
| **Marital duration (in years)** |  |  |
| 0-4 years | 1.00 | 1.00 |
| 5-9 years | 0.69 (0.41-1.16) | 0.75 (0.38-1.49) |
| 10-14 years | 0.60 (0.33-1.10) | 0.28 (0.12-0.64) |
| 15+ years | 0.51 (0.27-0.95) | 0.17 (0.05-0.55) |
| **Decision on women’s health care** |  |  |
| Respondent alone | 1.00 | 1.00 |
| Partner alone | 0.97 (0.53-1.79) | 1.11 (0.56-2.20) |
| Jointly | 0.76 (0.53-1.09) | 0.85 (0.57-1.27) |
| Someone else | 0.73 (0.31-1.72) | 0.59 (0.23-1.53) |
| **Currently working** |  |  |
| No | 1.00 | 1.00 |
| Yes | 0.69 (0.44-1.06) | 0.89 (0.54-1.46) |
| **Wealth quintile** |  |  |
| Poorest | 1.00 | 1.00 |
| Poorer | 1.02 (0.55-1.88) | 0.62 (0.33-1.14) |
| Middle | 1.07 (0.57-2.02) | 0.80 (0.46-1.41) |
| Rich | 1.22 (0.64-2.33) | 1.06 (0.58-1.94) |
| Richest | 1.13 (0.56-2.26) | 0.86 (0.44-1.67) |
| **Religion** |  |  |
| Hindu | 1.00 | 1.00 |
| Muslim | 1.25 (0.78-2.00) | 1.51 (0.95-2.41) |
| Others | 0.96 (0.46-2.00) | 1.93 (1.17-3.19) |
| **Caste** |  |  |
| Scheduled cases/tribes | 1.00 | 1.00 |
| Non scheduled cases/tribes | 1.02 (0.70-1.49) | 1.03 (0.69-1.54) |
| **Place of residence** |  |  |
| Urban | 1.00 | 1.00 |
| Rural | 1.35 (0.94-1.95) | 1.40 (0.95-2.07) |

| **Table T5. Results of multinomial logistic regression analysis showing adjusted relative risk ratio (RRR) for examining the association between experience of IPV and contraceptive use discontinuation among condom users, India, 2015-16** | | |
| --- | --- | --- |
|  | **Condom** | |
|  | **DWSIN** | **DDNFN** |
|  | **RRR (95% CI)** | **RRR (95% CI)** |
| **IPV in last 12-months** |  |  |
| No IPV | 1.00 | 1.00 |
| Physical IPV | 1.30 (0.67-2.55) | 1.11 (0.60-2.06) |
| Emotional IPV | 4.16 (1.59-10.90) | 0.98 (0.28-3.44) |
| Sexual IPV | 2.13 (0.28-16.14) | 0.07 (0.01-0.59) |
| Multiple forms of IPV | 1.13 (0.56-2.25) | 1.22 (0.63-2.36) |
| **Age of women** |  |  |
| 15-24 | 1.00 | 1.00 |
| 25-34 | 0.94 (0.51-1.73) | 1.58 (0.94-2.67) |
| 35+ | 0.74 (0.33-1.67) | 1.87 (0.81-4.28) |
| **Number of living children** |  |  |
| 0-1 | 1.00 | 1.00 |
| 2 | 1.07 (0.56-2.05) | 0.53 (0.26-1.10) |
| 3+ | 1.51 (0.64-3.53) | 0.64 (0.29-1.42) |
| **Duration of use before the observation period** |  |  |
| 1-12 months | 1.00 | 1.00 |
| 13-24 months | 0.33 (0.18-0.61) | 0.78 (0.44-1.40) |
| 25-36 months | 0.28 (0.13-0.61) | 0.67 (0.30-1.50) |
| 37+ months | 0.18 (0.10-0.34) | 0.35 (0.17-0.72) |
| **Women's schooling** |  |  |
| No schooling | 1.00 | 1.00 |
| Primary | 1.41 (0.65-3.08) | 0.91 (0.47-1.75) |
| Secondary | 0.96 (0.52-1.77) | 0.68 (0.37-1.24) |
| Higher | 0.79 (0.31-2.02) | 0.79 (0.33-1.90) |
| **Marital duration (in years)** |  |  |
| 0-4 years | 1.00 | 1.00 |
| 5-9 years | 0.84 (0.41-1.71) | 0.58 (0.21-1.60) |
| 10-14 years | 0.86 (0.38-1.95) | 0.33 (0.11-0.96) |
| 15+ years | 0.73 (0.29-1.83) | 0.19 (0.05-0.79) |
| **Decision on women’s health care** |  |  |
| Respondent alone | 1.00 | 1.00 |
| Partner alone | 1.20 (0.55-2.60) | 1.00 (0.43-2.31) |
| Jointly | 0.66 (0.40-1.08) | 0.71 (0.44-1.17) |
| Someone else | 0.62 (0.20-1.94) | 0.70 (0.26-1.90) |
| **Currently working** |  |  |
| No | 1.00 | 1.00 |
| Yes | 0.85 (0.46-1.58) | 1.18 (0.65-2.16) |
| **Wealth quintile** |  |  |
| Poorest | 1.00 | 1.00 |
| Poorer | 1.28 (0.51-3.18) | 0.57 (0.27-1.23) |
| Middle | 0.99 (0.41-2.40) | 0.54 (0.24-1.19) |
| Rich | 1.02 (0.41-2.56) | 0.92 (0.44-1.93) |
| Richest | 1.17 (0.46-2.97) | 0.68 (0.29-1.57) |
| **Religion** |  |  |
| Hindu | 1.00 | 1.00 |
| Muslim | 1.11 (0.61-2.02) | 1.12 (0.61-2.04) |
| Others | 0.92 (0.46-1.83) | 1.85 (0.95-3.61) |
| **Caste** |  |  |
| Scheduled cases/tribes | 1.00 | 1.00 |
| Non scheduled cases/tribes | 0.82 (0.47-1.44) | 1.01 (0.65-1.58) |
| **Place of residence** |  |  |
| Urban | 1.00 | 1.00 |
| Rural | 1.11 (0.71-1.72) | 1.44 (0.92-2.24) |
| Note. IPV: Intimate partner violence, DWSIN: Discontinuation while still in need, DDNFN: Discontinuation due to no further need | | |

| **Table T6.** Results of multinomial logistic regression analysis showing adjusted relative risk ratio (RRR) for examining the association between experience of IPV and contraceptive use discontinuation among pill users, India, 2015-16 | | |
| --- | --- | --- |
|  | **Pill** | |
|  | **DWSIN** | **DDNFN** |
|  | **RRR (95% CI)** | **RRR (95% CI)** |
| **IPV in last 12-months** |  |  |
| No IPV | 1.00 | 1.00 |
| Physical IPV | 0.56 (0.28-1.13) | 0.45 (0.19-1.05) |
| Emotional IPV | 0.39 (0.09-1.81) | 1.57 (0.50-4.88) |
| Sexual IPV | 0.80 (0.11-5.71) | 2.34 (0.50-11.07) |
| Multiple forms of IPV | 0.51 (0.22-1.18) | 0.91 (0.35-2.36) |
| **Age of women** |  |  |
| 15-24 | 1.00 | 1.00 |
| 25-34 | 1.06 (0.51-2.18) | 0.91 (0.40-2.06) |
| 35+ | 1.42 (0.60-3.31) | 0.79 (0.23-2.73) |
| **Number of living children** |  |  |
| 0-1 | 1.00 | 1.00 |
| 2 | 2.13 (1.04-4.39) | 0.54 (0.26-1.11) |
| 3 | 2.57 (1.06-6.25) | 0.88 (0.28-2.80) |
| **Duration of use before the observation period** |  |  |
| 1-12 months | 1.00 | 1.00 |
| 13-24 months | 0.87 (0.45-1.67) | 1.41 (0.62-3.24) |
| 25-36 months | 0.60 (0.24-1.48) | 0.80 (0.27-2.35) |
| 37+ months | 0.34 (0.18-0.65) | 1.27 (0.38-4.24) |
| **Women's schooling** |  |  |
| No schooling | 1.00 | 1.00 |
| Primary | 0.61 (0.27-1.41) | 0.96 (0.32-2.86) |
| Secondary | 1.12 (0.57-2.22) | 1.11 (0.49-2.51) |
| Higher | 0.69 (0.24-2.02) | 1.03 (0.30-3.54) |
| **Marital duration (in years)** |  |  |
| 0-4 years | 1.00 | 1.00 |
| 5-9 years | 0.47 (0.20-1.13) | 0.90 (0.34-2.40) |
| 10-14 years | 0.36 (0.14-0.93) | 0.20 (0.05-0.76) |
| 15+ years | 0.29 (0.10-0.84) | 0.13 (0.01-1.07) |
| **Decision on women’s health care** |  |  |
| Respondent alone | 1.00 | 1.00 |
| Partner alone | 0.46 (0.13-1.69) | 1.40 (0.20-1.07) |
| Jointly | 0.81 (0.46-1.43) | 1.02 (0.54-1.95) |
| Someone else | 0.81 (0.17-3.80) | * |
| **Currently working** |  |  |
| No | 1.00 | 1.00 |
| Yes | 0.81 (0.17-3.80) | 0.46 (0.20-1.07) |
| **Wealth quintile** |  |  |
| Poorest | 1.00 | 1.00 |
| Poorer | 0.62 (0.28-1.40) | 0.66 (0.27-1.61) |
| Middle | 1.04 (0.47-2.32) | 1.09 (0.50-2.34) |
| Rich | 1.59 (0.69-3.67) | 1.09 (0.38-3.12) |
| Richest | 1.24 (0.48-3.16) | 1.24 (0.40-3.83) |
| **Religion** |  |  |
| Hindu | 1.00 | 1.00 |
| Muslim | 1.26 (0.66-2.38) | 1.85 (0.86-3.94) |
| Others | 1.17 (0.36-3.84) | 2.09 (1.05-4.16) |
| **Caste** |  |  |
| Scheduled caste/tribes | 1.00 | 1.00 |
| Non-scheduled caste/tribes | 1.30 (0.73-2.33) | 0.89 (0.43-1.82) |
| **Place of residence** |  |  |
| Urban | 1.00 | 1.00 |
| Rural | 1.46 (0.74-2.86) | 1.52 (0.71-3.28) |
| Note. * Insufficient event of DDNFN occurred among those who reported that decision on women’s health care is taken by someone else.  IPV: Intimate partner violence, DWSIN: Discontinuation while still in need, DDNFN: Discontinuation due to no further need | | |
